# Supplementary material for: The Construction and Exploration of a Comprehensive MicroRNA Centered Regulatory Network in Foxtail Millet (Setaria italica L.)
Source: Front Plant Sci. 2022 May 6;13:848474. doi: 10.3389/fpls.2022.848474 (PMC9121102; doi:10.3389/fpls.2022.848474)
Supplement: Supplementary file 7 [file Data_Sheet_7.zip › Sit-miR529a_Seita.7G241300.1_2732_TPlot.pdf]

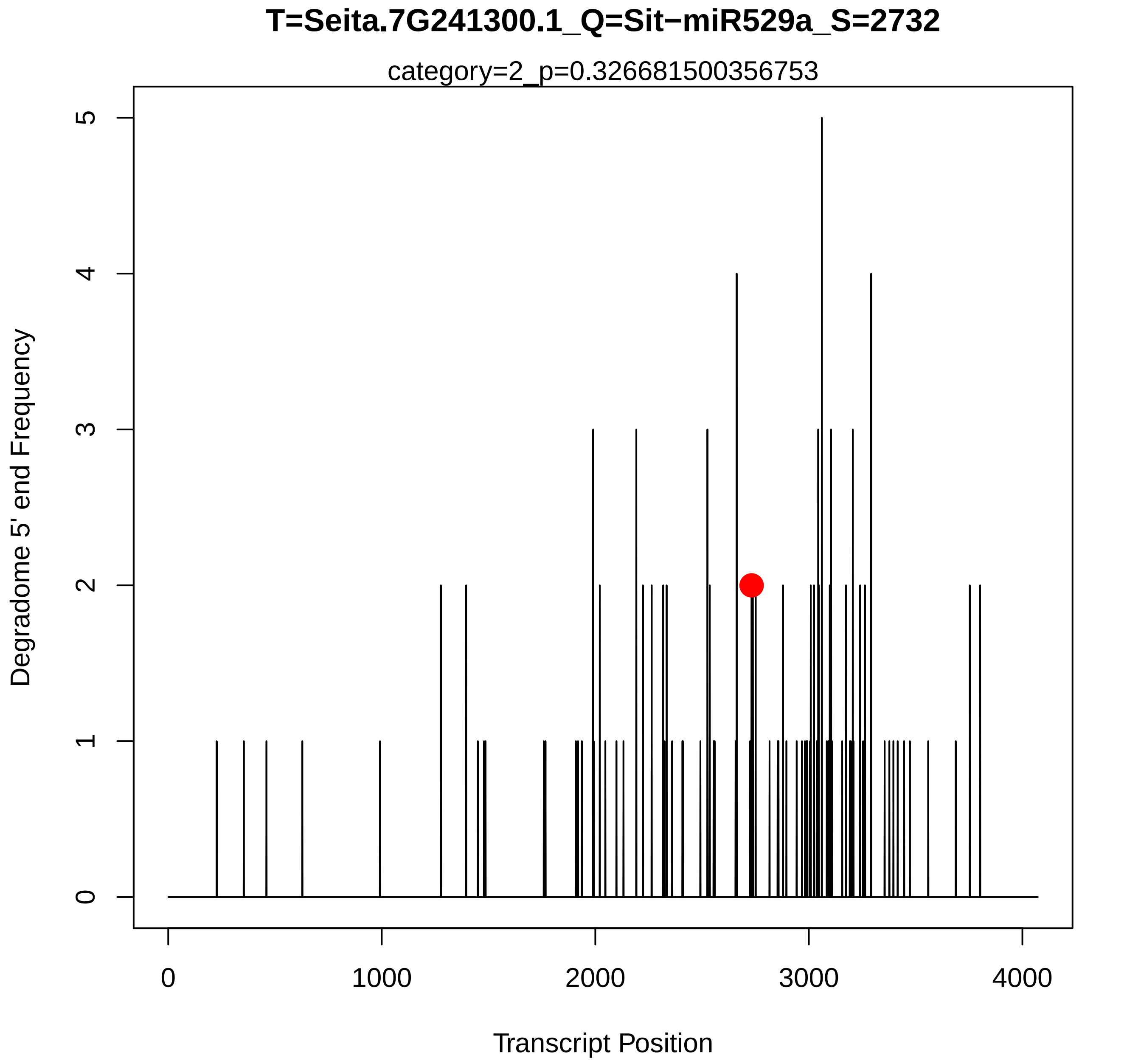

T=Seita.7G241300.1_Q=Sit−miR529a_S=2732
category=2_p=0.326681500356753
Degradome 5' end Frequency
Transcript Position
0
1
2
3
4
5
0
1000
2000
3000
4000
